# Developing and validating a national set of standards for undergraduate medical education using the WFME framework: the experience of an accreditation system in Iran

**DOI:** 10.1186/s12909-023-04343-9

**Published:** 2023-05-24

**Authors:** Roghayeh Gandomkar, Tahereh Changiz, Athar Omid, Mahasti Alizadeh, Majid Khazaei, Abtin Heidarzadah, Pouria Rouzrokh, Mitra Amini, Hamid Honarpisheh, Reza Laripour, Farshid Abedi, Babak Sabet, Azim Mirzazadeh

**Affiliations:** 1 Department of Medical Education, School of Medicine, Tehran University of Medical Sciences, Tehran, Iran; 2 Health Profession Education Research Center, Tehran University of Medical Sciences, Tehran, Iran; 3 Department of Medical Education, Isfahan University of Medical Sciences, Isfahan, Iran; 4 Medical Education Research Center, Isfahan University of Medical Sciences, Isfahan, Iran; 5 Social Determinants of Health Research Center, Tabriz University of Medical Sciences, Tabriz, Iran; 6 Medical Education Research Center, Tabriz University of Medical Sciences, Tabriz, Iran; 7 Department of Physiology, Mashhad University of Medical Sciences, Mashhad, Iran; 8 Medical Education Research center, Guilan University of Medical Sciences, Guilan, Iran; 9 Artificial Intelligence Laboratory, Department of Radiology, Mayo Clinic, Rochester, MN USA; 10 Clinical Education Research Center, Shiraz University of Medical Sciences, Shiraz, Iran; 11 Deputy of Education Faculty Member, Ministry of Health and Medical Education, Secretariat of the Council of Undergraduate Medical Education, Tehran, Iran; 12 Department of Social and Preventive Medicine, School of Medicine, Aja University of Medical Sciences, Tehran, Iran; 13 Department of Infectious Diseases, School of Medicine, Infectious Diseases Research Center, Birjand University of Medical Sciences, Birjand, Iran; 14 Department of Surgery, Faculty of Medicine, Shahid Beheshti University of Medical Sciences, Tehran, Iran; 15 Health Profession Education Research Center, Tehran University of Medical Sciences, No. 57, Hojjatdust Alley, Naderi St., Keshavarz Blvd, Tehran, 141663591 Iran; 16 Department of Internal Medicine, School of Medicine, Tehran University of Medical Sciences, Tehran, Iran

**Keywords:** Standard, Undergraduate medical education, WFME

## Abstract

**Background:**

Defining standards is the first step toward quality assurance and improvement of educational programs. This study aimed at developing and validating a set of national standards for the Undergraduate Medical Education (UME) program through an accreditation system in Iran using the World Federation for Medical Education (WFME) framework.

**Methods:**

The first draft of standards was prepared through consultative workshops with the participation of different UME program stakeholders. Subsequently, standards were sent to medical schools and UME directors were asked to complete a web-based survey. The content validity index at the item level (I-CVI) was computed using criteria including clarity, relevance, optimization and evaluability for each standard. Afterward, a full-day consultative workshop was held and a wide range of UME stakeholders across the country (n = 150) discussed the survey results and made corrections to standards.

**Results:**

Analysis of survey results showed that relevance criteria had the best CVI as only 15 (13%) standards demonstrated CVI < 0.78. More than two-thirds (71%) and a half (55%) of standards showed CVI < 0.78 for optimization and evaluability criteria. The final set of UME national standards was structured in 9 areas, 24 sub-areas, 82 basic and 40 quality development standards, and 84 annotations.

**Conclusions:**

We developed and validated national standards as a framework to ensure the quality of UME training with input from UME stakeholders. We used WFME standards as a benchmark while addressing local requirements. The standards and participatory approach to developing standards may guide relevant institutions.

**Supplementary Information:**

The online version contains supplementary material available at 10.1186/s12909-023-04343-9.

## Introduction

There has been a growing interest, during the past decades, in improving medical education by developing and adopting standards and guidelines [1]. Academic institutions tend to employ existing standards as a framework to inform the design of educational programs in terms of content and process [2], and to direct the self-evaluation of the programs in respect of strengths, weaknesses and needs for improvement [3]. For example, Allen et al. (2022) explored the applicability of Liaison Committee on Medical Education (LCME) accreditation standards to evaluate the doctor of medicine (MD) degree program at Khalifa University in the United Arab Emirates [4]. The existence of agreed-upon standards may lead to consistency and convergence between programs in different institutions as they attempt to meet the standards [5]. Furthermore, external regulatory authorities such as accreditation agencies generally set and use standards for reviewing the programs’ suitability, ensuring a minimum level of program quality and for encouraging improvements beyond the levels indicated [6, 7]. For instance, the National Commission for Academic Assessment and Accreditation (NCAAA) stablished in Saudi Arabia [8], implemented accreditation of undergraduate programs in health science education and examined its impact on educational processes [9, 10]. Institute of Health Professions Education and Research (IHPER) is a local accreditation agency with the aim of setting standards and implementing the accreditation process for medical education in Pakistan [11]. Residency programs in Japan are accredited by governmental bodies which use standards congruent with Accreditation Council for Graduate Medical Education (ACGME) Common Program Requirements [12]. The external standards will also inform students of what is expected of them and what they could expect from their program. Finally, standards provide students, patients and health service employers with reassurance that the quality of training has been satisfactory [13, 14]. In this regard, Goroll et al. (2014) provided a new framework for the accreditation of residency Programs in internal medicine whereby accreditation moves from an external audit of the educational process to continuous assessment and improvement [15].

The Joint Committee on Standards for Educational Evaluation (2010) defined an evaluation standard as a “principle mutually agreed to by people engaged in a professional practice that if met, will enhance the quality and fairness of that professional practice” [16]. In this regard, the World Federation for Medical Education (WFME) developed a series of international standards for quality improvement of medical education in 2003 [6]. The aim was to offer a basis for medical schools, organizations, and authorities responsible for quality assurance throughout all three phases of medical education including Basic (undergraduate) Medical Education (BME) [17], Postgraduate Medical Education (PME) [18] and Continuing Professional Development (CPD) [19]. The series was updated in 2015, after an initial revision of the BME standards in 2012 [20]. The WFME standards were widely adopted at national and regional levels, sometimes with the consideration of local adaptations [21–24]. In particular, the Association for Medical Education in the Western Pacific Region (AMEWPR), has since aligned its standards and guidelines more thoroughly to those of WFME, along with regional specifications [25]. Sjostrom et al. (2019) reviewed the application of the WFME standards and found that 29 papers reported the use of these standards including as guidelines for quality improvement, and in the evaluation of programs and accreditation of medical schools. Based on their results, three studies employed the WFME framework for standard development. The authors concluded that WFME standards may serve as a template for developing standards while addressing local specifications [2].

Accreditation is a formal professional review process whereby an organization grants approval of educational programs or institutions heavily relying on experts’ judgment. Accreditation consists of five major elements: (1) existing structure (an independent or governmental, and regional or national organization), (2) standards developed and published by the accreditation agency, (3) specified schedule (e.g. review educational programs every 5 years), (4) opinions of multiple experts in the form of commissions or committees for decision making, and (5) status of educational programs or institutions affected by results. The process of accreditation begins with a self-study whereby the institution investigates how well its educational program has met the standards of the accrediting body. Afterward, a site visit of the program is conducted by a group of experts. The site visit report is then reviewed by another group of experts in the form of a standing commission or committee and a decision is made which will be provided to the institution [26].

Accreditation standards are developed as general and global statements which arise some concerns about their evaluability. Hence, different agencies have tried making clarification by adding intents or annotations to explain the standards and questions, performance indicators or sample evidence to guide the review process. Although evaluation in accreditation is mainly based on expert opinions, they use qualitative evidence such as the interview with teachers as well as quantitative data like the achievement of program learning outcomes (knowledge, skills and attitudes) or competence by students [27].

Health professional education accreditation systems generally differ in terms of standards taxonomies (types and levels). Types of criteria included in the standards may be structures, processes or outcomes, or mix of these criteria. Level of expectations can be set at the minimum or aspirational levels, and in some cases, a mixed model may be used. In terms of processes for the development and renewal of accreditation standards, accreditation systems commonly recruit consensus-based approaches to receive input from local experts while integrating areas of innovation from other systems. This approach can help to ensure better face validity of standards and acceptance among stakeholders of the educational program [28].

Medical education witnessed a substantial rise in its UME programs and medical student admissions during the 80 and 90 s in Iran which challenged the quality of MD training [29]. Consequently, the Secretariat of the Council for Undergraduate Medical Education (SCUME) was formed under the governance of the Ministry of Health and Medical Education (MoHME) as a structure responsible for ensuring the quality of 63 Undergraduate Medical Education (UME) programs throughout country [30]. Over the last two decades, SCUME has been involved in several activities to promote the quality of UME programs and to resolve the traditional curriculum issues [31, 32]. For instance, the competency framework for the UME program was formulated and approved in 2017. Consequently, the national UME curriculum was revised and medical schools are requested to update their UME programs based on the new curriculum and competency framework. Nevertheless, few medical schools undertook a full reform in their program [33, 34] and several others initiated some aspects of the new curriculum including the incorporation of integrations, utilization of student-centered teaching methods and interactive techniques and renewal of assessment procedures [35, 36]. To ensure the quality of UME programs and accelerate these initiatives SCUME started the implementation of an accreditation system in September 2017. Developing standards is the first and most important step in implementing an accreditation system [26]. Although a set of standards had been developed by SCUME in 2007, it was not representative of recent changes and innovations in the field of UME at the international and national levels. This study aimed to develop and validate a set of national standards for the UME program through our accreditation system using the WFME framework. The results of this study guide our UME program directors for quality actions and function as a basis for the accreditation system. Furthermore, since UME accreditation was the first experience of an accreditation system for educational programs in Iran, the standard set can inform subsequent accreditation systems.

## Materials and methods

This study was performed between October 2016 and July 2017 at SCUME in Iran. We followed a consultative approach to develop and validate a set of standards involving various groups of UME program stakeholders. Initially, a taskforce was established at SCUME with the responsibility of overseeing the definition of standards. Task force members agreed on using the latest version of WFME BME standards (revised in 2015) as a starting point for drafting the standards in terms of both content and structure after reviewing the standard set of several regulatory authorities for UME as it deemed most suited to our UME context. The WFME BME set of standards comprises 106 basic (minimum) standards, 90 quality development (aspirational) standards and 127 annotations organized into 9 areas with a total of 35 sub-areas. Nine areas are ‘Mission and Objectives’, ‘Educational Program’, ‘Assessment of Students’, ‘Students’, ‘Academic Staff/Faculty’, ‘Educational Resources’, ‘Program Evaluation’, ‘Governance and Administration’ and ‘Continuous Renewal’. Sub-areas define specific aspects of an area and annotations provide clarification to the standards [37].

Eight working groups were formed under the supervision of the task force, each responsible for developing one standard area. Each working group consisted of 3 to 4 members and was selected on basis of their expertise and experience in the standard area and with geographical coverage across the country. Members of the working group were mainly faculty who had teaching experience for medical students, and visiting and evaluating UME programs. Some of them had experience in administrative work in related areas (e.g. being the vice dean for administrative and financial affairs for ‘Educational Resources’ or being the director of clerkship for ‘Educational Program’). A medical student member was considered for working groups in areas such as ‘Students’ or ‘Educational Resources’ that needed student input. We have a pool of students and graduates of medical education (MSc and PhD) in Iran [38, 39]. Therefore, we included at least one member among them for each working group to guide the group regarding medical education concepts. Since the content of standards of area nine (i.e. Continuous Renewal) was related to other areas, its writing was postponed to the final phase.

**Fig. 1:**
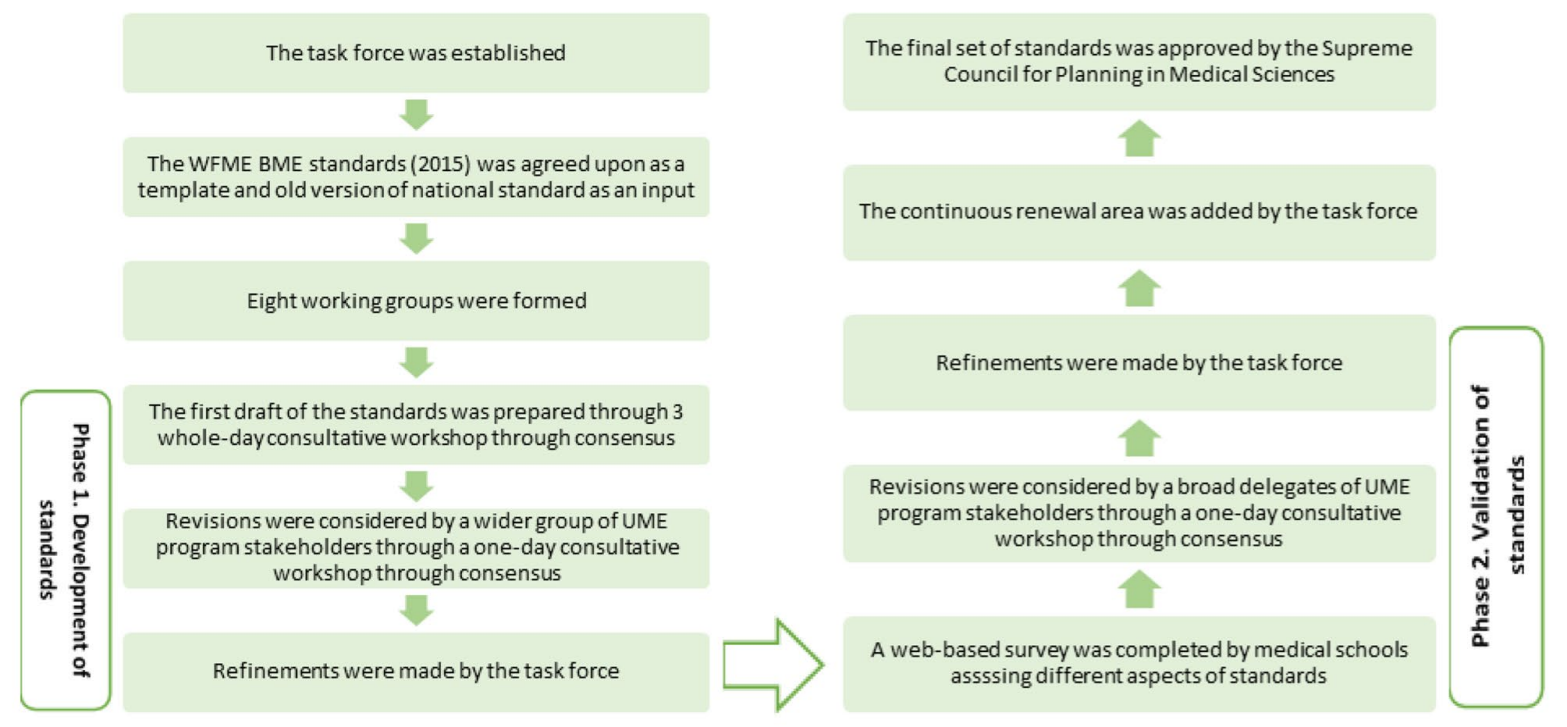
Phases and steps of developing the set of national standards

We followed two main phases: development and validation of standards [Fig. 1].

### Phase 1. Development of standards

The first draft of standards was prepared through three full-day consultative workshops. In each working group, members discussed each WFME standard and decided to include (with or without revision) or ignore it concerning the local UME specifications. A member of the group was responsible to write down the agreed-upon standard in Farsi. The previous version of the national standards was available for working groups as supplementary material. After 4 h of working on standards, each working group presented its proposed standards, and then members of other working groups provided their suggestions. It took 2.5 h for each area on average. Two members of the task force (TCH & AM) who had extensive experience in discussion facilitation for the group involving diverse stakeholders facilitated the discussions and one (RG) took notes. A brief guide was provided to each group at the beginning consisting of tips on reading each standard several times and discussing it in-depth in the group, and sharing with the large group if there were areas of disagreement or concerns. Standards were later modified based on the provided comments in several task force meetings. All working groups’ presentations had been recorded and were used for further refinements if notes were not complete.

In the next step, a full-day consultative workshop was held with the participation of members of the Board of UME Examiners and Board of Health Professions Education Examiners along with working groups members. Affiliated members of the boards were assigned to working groups to include their inputs on the second version of standards. A similar procedure to the previous meeting was followed. Finally, the proposed set of standards was examined in terms of content (overlaps between areas and completeness of each area) and writing format and refinements were made by the task force [Fig. 1].

### Phase 2. Validation of standards

The set of standards developed in phase 1 was considered for the validation study. First, a survey was developed with six questions for each standard. Four yes/no questions were asked to determine the clarity (is the standard clear?), relevance (is the standard relevant?), optimization (is the standard optimum?) and evaluability (is the standard evaluable?) of the standard. A two-option question was added related to the level of the standards (i.e. should the standard be considered as a basic or quality improvement?) and an open-ended question for further comments. There was also a box at the end of each standard area to provide additional suggestions for the area as a whole. The web-based survey was sent to 49 public medical schools via formal correspondence by SCUME and UME directors were asked to complete the survey. The directors were encouraged to complete the survey after obtaining other UME stakeholders in their institutions.

Completed surveys underwent quantitative and qualitative analyses. For quantitative analysis, the content validity index at the item level (I-CVI) was computed using Microsoft Excel as the number of all respondents divided by several medical schools that agreed a standard was a good fit to a criterion (clarity, relevance, optimization or evaluability) [40]. For two-option questions (basic or quality improvement), the percent of responses consistent with the preassigned standard level was computed. All responses to open-ended questions were summarized and categorized by standards, sub-areas and areas. No standards were removed in this step and the results of the analyses were used as a trigger for further expert discussion.

In the next step, a full-day consultative workshop was held and UME stakeholders across the country were invited. Stakeholders included deans of the medical schools, Associate deans of UME, directors of education development centers (EDC) and School of Medicine Education Development Offices (EDO), experts in the field of medical education including medical education students or graduates, experts involved in UME training and evaluation, faculty members and medical students with coverage all UME programs across the country. We recruited a diverse group of stakeholders to make a balance between the perspectives and alleviate the natural desire of medical school administrations to downgrade standards. Participants were divided into 6 groups compatible with the standards area. The ‘Assessment of Students’ and ‘Program Evaluation’ as well as ‘Governance and Administration’ and ‘Mission and Objectives’ areas which had fewer standards and were related conceptually were assigned to one group. There were representatives from each stakeholder cluster in groups. One hundred and fifty people participated in the workshop with 25 people in each group on average. The quantitative and qualitative results of the survey were reviewed for each standard throughout the group work. If the I-CVI was more than 0.78, experts maintained the standards and minor refinements were made if there were any written comments. If the I-CVI was less than 0.78 for any of the criteria, experts discussed the standards and made major corrections and revisions with the consideration of written comments [40]. Finally, if the revision was impossible, the standard was deleted. For two-option questions, if there were ≥ 70 agreements with the preassigned standard level, the category was confirmed.

In the end, task force members reviewed the set of standards for coherency and consistency and writing issues and final refinements were considered. The ‘Continuous Renewal’ area was added and the final set of standards was approved by the Supreme Council for Planning in Medical Sciences [Fig. 1].

## Results

The draft version of the standards comprises 73 basic and 38 quality improvement standards, and 79 annotations (190 in total). Twenty-eight medical schools (response rate = 57%) completed the validation survey. Table 1 contains I-CVIs for clarity, relevance, optimization and evaluability criteria and percent agreements for the standard level criterion by standards. Relevance and clarity criteria had the best I-CVIs as only 15 (13.51%) and 17 (15.31%) standards demonstrated CVI < 0.78, respectively. More than two-thirds (71.17%) and a half (55.85%) of standards showed CVI < 0.78 for optimization and evaluability criteria, respectively. For 25 (22.52%) standards, all criteria were more than 0.78 and for 8 (7.2%) standards, all criteria were less than 0.78. The ‘Instructional Program’ and ‘Program Evaluation’ were areas with most standards with unsatisfactory criteria. Finally, 86 (77.47%) standards showed good agreement in terms of quality levels.

**Table 1:** I-CVIs for clarity, relevance, optimization and evaluability criteria and percent agreements for a standard level criterion by standards

Standards	Clarity	Relevance	Optimization	Evaluability	Basic or QI?	Standards	Clarity	Relevance	Optimization	Evaluability	Basic or QI?	Standards	Clarity	Relevance	Optimization	Evaluability	Basic or QI?	
B1-1-1	0.82	0.82	0.75	0.71	89	QI4-1-1	0.89	0.89	0.64	0.71	93	QI6-3-1	0.82	0.75	0.71	0.71	57	
B1-1-2	0.86	0.82	0.68	0.75	86	QI4-1-2	0.79	0.82	0.68	0.75	79	QI6-3-2	0.89	0.82	0.79	0.82	82	
B1-1-3	0.89	0.82	0.75	0.64	86	B4-2-1	0.93	0.89	0.75	0.62	86	B6-4-1	0.79	0.82	0.71	0.79	93	
QI1-1-1	0.86	0.75	0.46	0.54	75	B4-2-2	0.96	0.89	0.71	0.82	89	B6-4-2	0.82	0.93	0.75	0.82	96	
QI1-1-2	0.82	0.86	0.57	0.61	75	B4-2-3	0.82	0.82	0.68	0.64	75	B6-4-3	0.82	0.86	0.79	0.71	93	
B1-2-1	0.64	0.71	0.46	0.54	71	B4-2-4	0.79	0.79	0.57	0.71	79	B6-4-4	0.89	0.82	0.75	0.71	79	
B2-1-1	0.82	0.82	0.75	0.75	89	B4-2-5	0.79	0.82	0.71	0.64	86	QI6-4-1	0.68	0.79	0.68	0.61	89	
B2-1-2	0.46	0.71	0.54	0.46	71	B4-2-6	0.89	0.82	0.79	0.86	68	B6-5-1	0.71	0.79	0.71	0.64	82	
B2-1-3	0.75	0.75	0.71	0.50	79	B4-2-7	0.89	0.82	0.68	0.71	64	B6-5-2	0.89	0.93	0.75	0.79	71	
QI2-1-1	0.75	0.86	0.71	0.64	36	QI4-2-1	0.89	0.82	0.75	0.71	75	QI6-5-1	0.82	0.93	0.75	0.75	53	
B2-2-1	0.75	0.82	0.71	0.75	86	QI4-2-2	0.89	0.86	0.71	0.79	68	QI6-5-2	0.82	0.86	0.64	0.68	86	
B2-2-2	0.89	0.86	0.75	0.75	89	QI4-2-3	0.86	0.86	0.75	0.79	79	B6-6-1	0.89	0.82	0.71	0.75	75	
B2-2-3	0.75	0.64	0.57	0.61	75	B4-3-1	0.79	0.82	0.68	0.68	64	QI6-6-1	0.82	0.79	0.64	0.71	79	
B2-2-4	0.79	0.86	0.71	0.71	79	QI4-3-1	0.75	0.82	0.64	0.71	68	B7-1-1	0.82	0.89	0.71	0.86	82	
QI2-2-1	0.75	0.82	0.64	0.54	54	B5-1-1	0.86	0.86	0.79	0.75	89	B7-1-2	0.86	0.89	0.79	0.79	82	
B2-3-1	0.71	0.75	0.64	0.71	54	B5-1-2	0.86	0.86	0.75	0.86	93	B7-1-3	0.86	0.89	0.75	0.75	82	
B2-3-2	0.93	0.82	0.79	0.79	82	B5-1-3	0.96	0.89	0.82	0.79	89	QI7-1-1	0.79	0.82	0.68	0.71	71	
QI2-3-1	0.71	0.82	0.54	0.68	71	B5-1-4	0.96	0.89	0.75	0.82	89	B7-2-1	0.64	0.64	0.68	0.64	75	
QI2-3-2	0.82	0.71	0.68	0.79	71	QI5-1-1	0.93	0.82	0.71	0.82	61	QI7-2-1	0.64	0.71	0.39	0.43	89	
QI2-3-3	0.82	0.82	0.64	0.50	46	QI5-1-2	0.86	0.75	0.61	0.68	75	QI7-2-2	0.61	0.64	0.46	0.43	82	
B2-4-1	0.93	0.86	0.82	0.86	96	B5-2-1	0.96	0.86	0.75	0.82	93	QI7-2-3	0.82	0.82	0.79	0.79	82	
B2-4-2	0.86	0.82	0.75	0.71	79	B5-2-2	0.86	0.86	0.79	0.68	89	B7-3-1	0.82	0.86	0.75	0.79	82	
B2-4-3	0.89	0.86	0.75	0.79	93	B5-2-3	0.75	0.82	0.68	0.64	86	QI7-3-1	0.79	0.79	0.61	0.71	82	
B2-4-4	0.86	0.86	0.75	0.79	89	B5-2-4	0.86	0.86	0.64	0.71	75	QI7-3-2	0.82	0.79	0.64	0.68	79	
QI2-4-1	0.93	0.86	0.79	0.79	32	B5-2-5	0.93	0.89	0.75	0.71	79	B8-1-1	0.93	0.86	0.79	0.86	93	
B3-1-1	0.96	0.89	0.82	0.86	96	B5-2-6	0.89	0.86	0.79	0.64	82	B8-1-2	0.93	0.82	0.79	0.86	96	
B3-1-2	0.93	0.86	0.79	0.68	89	B5-2-7	0.89	0.75	0.64	0.64	75	QI8-1-1	0.75	0.86	0.71	0.79	61	
B3-1-3	0.86	0.89	0.75	0.79	86	QI5-2-1	0.93	0.86	0.75	0.82	36	QI8-1-2	0.89	0.89	0.71	0.82	61	
B3-1-4	0.93	0.86	0.82	0.82	86	B6-1-1	0.86	0.86	0.79	0.82	96	B8-2-1	0.89	0.86	0.75	0.86	93	
B3-1-5	0.82	0.89	0.71	0.75	64	B6-1-2	0.86	0.89	0.79	0.82	93	QI8-2-1	0.82	0.86	0.68	0.71	50	
B3-1-6	0.86	0.93	0.86	0.86	68	QI6-1-1	0.93	0.89	0.71	0.79	57	B8-3-1	0.93	0.89	0.79	0.86	96	
QI3-1-1	0.89	0.89	0.79	0.82	32	B6-2-1	0.93	0.89	0.79	0.79	89	B8-3-2	0.93	0.89	0.79	0.86	93	
QI3-1-2	0.93	0.93	0.82	0.86	54	QI6-2-1	0.89	0.82	0.64	0.64	64	B8-4-1	0.82	0.93	0.82	0.79	86	
QI3-1-3	0.82	0.86	0.82	0.75	71	B6-3-1	0.89	0.86	0.75	0.79	79	B8-4-2	0.82	0.82	0.71	0.75	68	
B4-1-1	0.82	0.75	0.61	0.57	68	B6-3-2	0.86	0.89	0.75	0.82	79	B8-4-3	0.89	0.86	0.79	0.82	89	
B4-1-2	0.89	0.89	0.71	0.86	93	B6-3-3	0.96	0.89	0.75	0.82	86	QI8-4-1	0.61	0.75	0.68	0.61	75	
B4-1-3	0.96	0.89	0.86	0.82	89	B6-3-4	0.86	0.75	0.71	0.79	79	B8-5-1	0.82	0.82	0.75	0.68	75	

I-CVI = content validity index at item level, QI = quality improvement, B = basic

Colored boxes indicate I-CVIs < 0.78 or agreements < 70%

1-1-1 stands for area 1-subarea 1-standard 1

We received 633 written comments (3.33 per standard or annotation) by validation survey which was compatible with the validity criteria. Some comments pointed to two criteria so, we labeled 657 validity criteria in total. Table 2 presents numbers and examples of comments for standards by areas and types of validity criteria. As can be seen, most comments (n = 465, 70.77%) were related to clarity which referred to editing (e.g. *it is better to mention health problems instead of the phrase ‘transnational aspects of health*), the content of standards (e.g. *It is necessary to prepare a printed and electronic handbook for students and provide it to newcomers)*, or lack of clarity (e.g. The research infrastructure is unclear). Few comments (n = 11, 1.67%) were connected with relevance (e.g. This standard can be contrary to the missions of medical schools). We indicated a total of 85 (12.93%) comments for optimizations (e.g. *usually, the conditions for using various assessment methods are not provided*), 34 (5.17%) for evaluability (e.g. Two items “adequate number and variety of patients” and “supervision of clinical training” cannot be evaluated) and 49 (7.45%) for the quality level of standard (e.g. These are as the requirements for medical school).

**Table 2:** Numbers and examples of comments provided for standards by areas and validity criteria * Some comments in this area pointed to two criteria

Areas	comments (n)							Comments (examples)
	Clarity	Relevance	Optimization	Evaluability	Basic or QI?	Unrelated	Total	
1. Mission and Objectives	41	4	4	3	-	1	53	- It is better to mention ‘health problems’ instead of the phrase 'transnational aspects of health’. (clarity, QI1-1-2) - Given the difference in mission statements of universities, this standard can be contrary to the goals of universities. (relevance, QI1-1-2) - Standards of this area should be formulated based on the local characteristics of each university. (optimization, area) - Standard has been written in a general statement and it is not evaluable. (evaluability, B1-1-3)
2. Instructional Program	104	2	25	12	20	2	159*	- Religion, creed and race should be considered in the definition of educational justice. (clarity, B2-1-3) - There is no need for complementary and alternative medicine for the general practitioner. (relevance, B2-2-3) - This standard is applicable if the geographical and demographic conditions and facilities of the region allow. (optimization, B2-4-3) - How is the occurrence of competency-based education measured? (evaluability, B2-1-2)
3. Assessment of Students	27	-	8	3	5	-	40*	- The purpose of the summative assessment should be explained in detail. (clarity, B3-1-4) - Usually, the conditions for using various assessment methods are not provided. (optimization, B3-1-3)Provision of requirements and conditions for improving student learning is ambiguous and cannot be evaluated. (evaluability, OI3-1-3)
4. Students	58	-	19	3	4	6	89*	- It is necessary to prepare a printed and electronic handbook for students and provide it to newcomers. (clarity, B4-1-3) - The standard is not applicable since the admission process is centralized. (optimization, QI4-1-1)The type of students’ characteristics that are going to be surveyed studied by should be specified; Based on that, its evaluability can be determined. (evaluability, B4-1-1)
5. Academic Staff/Faculty	57	1	13	5	2	1	77*	- Delete the word ' selection’. (clarity, QI5-1-1) - It is mostly at the disposal of the medical school. (relevance, B5-1-1) - Considering the cultural characteristics of universities, it cannot be implemented everywhere (optimization, B5-2-4)It is difficult to measure the two verbs, familiarity and paying enough attention. (evaluability, B5-2-6)
6. Educational Resources	80	2	10	5	10	1	101*	- Does the ‘covered population’ refer to the needs of students or the needs of society? (clarity, QI6-2-1) - This standard is the mission of the university, not the medical school. (relevance, QI6-2-1) - Medical schools must be independent in terms of financial resources so that this standard can be implemented (optimization, B6-4-2)Two items "adequate number and variety of patients" and "supervision of clinical training" cannot be evaluated. (evaluability, B6-2-1)
7. Program Evaluation	50	2	2	-	3	-	57	- It is better to change the order of B7-1-1 and B7-1-2. (clarity, B7-1-1) - This standard is useless as long as student admission is concentrated. (relevance, B7-2-2) - In order to fulfill this standard, specialized counseling centers must be located in the medical school (optimization, B7-2-2)
8. Governance and Administration	40	-	2	2	5	1	48*	- It is better to change the order of B7-1-1 and B7-1-2. (clarity, B7-1-1) - Due to the different missions of our university, this standard has limited implementation (optimization, B8-5-1)It is difficult to measure this standard. (evaluability, B8-5-1)
Set of standards (overall)	8	-	2	1	1	1	9*	- The accountability of medical school to the community has less been emphasized in these standards and is reduced to the participation of stakeholders. (clarity) - Expectations from medical schools are set at the level of the university president and in some cases at the level of the ministry. (optimization) - The standards are developed as global statement and do not include numerical or quantitative indicators that stabilize them. (evaluability)
Total	465	11	85	34	49	13	633*	-
* Some comments in this area pointed to two criteria.								

Online Supplemental Appendix 1 contains the final set of UME national standards which were structured in nine areas similar to WFME BME standards, 24 sub-areas, 82 basic standards, 40 quality development standards and 84 annotations.

**Fig. 2:**
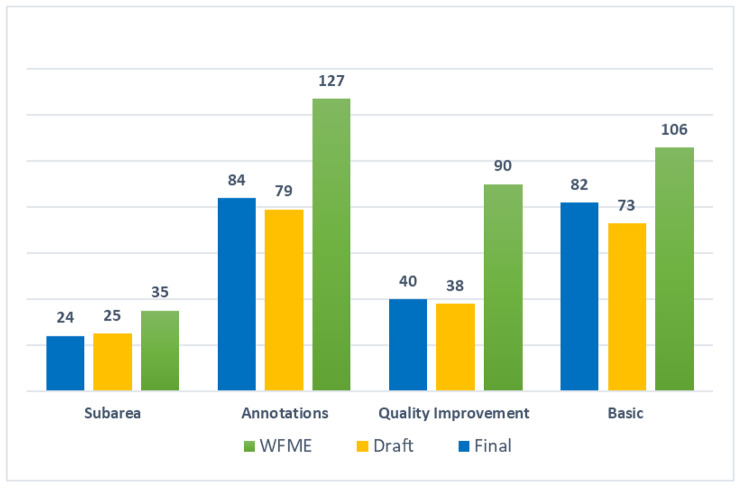
A total number of standard set components for WFME BME standards, and draft and final versions of national standards

Table 3 maps the number of standard set components (i.e. sub-areas, basic standards, quality improvement standards and annotations) per area for WFME BME standards, and draft (pre-validation) and final versions of national standards. Figure 2. Depicts the total number of standard set components for the three sets of standards. As can be seen, the total number of all components is lower in two versions of national standards compared with WFME standards. This reduction is more evident in quality improvement standards (WFME = 90, national = 40). Interestingly, the number of basic standards in three areas (i.e. Academic Staff/Faculty, Educational Resources, and Governance and Administration) increased in national standards.

**Table 3:** Number of standard set components per area for WFME BME standards, and draft and final versions of national standards

Areas	Sub-areas (n)			Basic standards (n)			Quality improvement standards (n)			Annotations (n)			
	WFME	Draft	Final	WFME	Draft	Final	WFME	Draft	Final	WFME	Draft	Final	
1. Mission and Objectives	4	2	2	19	4	4	8	2	2	20	9	10	
2. Educational Program	8	4	4	21	13	14	19	6	5	31	15	20	
3. Assessment of Students	2	0	0	10	6	7	5	3	3	8	6	9	
4. Students	4	3	3	13	11	11	7	6	6	12	10	11	
5. Academic Staff/Faculty	2	2	2	8	11	11	4	3	2	11	7	7	
6. Educational Resources	6	6	6	15	14	17	14	8	6	13	15	12	
7. Program Evaluation	4	3	2	10	5	5	13	6	3	13	7	6	
8. Governance and Administration	5	5	5	7	9	10	8	4	2	18	10	8	
9. Continuous Renewal	0	-	0	3	-	3	12	-	11	1	-	1	

## Discussion

This study aimed at developing and validating a set of national standards for the UME program using the WFME framework as an operating point to develop an accreditation system. The paper describes the process of standards definition as well as the results of translating WFME standards into the context of a developing country. Addressing the diversity of UME programs across the country, standards were drafted and iteratively refined and validated based on the inputs from different stakeholder groups using a survey and expert meetings. The development phase resulted in 73 basic and 38 quality improvement standards, and 79 annotations. Throughout the validation survey, relevance and clarity criteria showed acceptable I-CVIs while optimization and evaluability criteria demonstrated unsatisfactory I-CVIs. Interestingly, most written comments (70.77%) were related to clarity and a few (1.67%) were connected with relevance. A total of 12.93% and 5.17% of comments were considered optimizations and evaluability, respectively. After refinement based on the survey results and discussions in the consultative workshop, the final set of UME national standards was structured in nine areas similar to WFME BME standards, 24 sub-areas, 82 basic standards, 40 quality development standards and 84 annotations. The total number of all components was lower in the two versions of national standards compared with WFME standards. This reduction was more evident in quality improvement standards.

The validity of standards is conventionally examined by relevance (or importance) and clarity. Of the several identified studies on the validity of accreditation standards [41–43], all reported similar results to our study. They observed satisfactory results for both importance and clarity criteria yet, the ratings for clarity were lower than the ratings for importance. To the best of our knowledge, we did not find studies that evaluated the optimization and evaluability of accreditation standards. This is while defining optimal standards is a challenging task for accreditation agencies particularly those that operate in heterogeneous contexts [2]. Aligning with that, there are 63 UME programs in Iran that differ in terms of community to which healthcare services are provided, accessibility to resources, educational programs and environment, missions and objectives, student characteristics and quality of training provided [44–46]. Furthermore, various stakeholder groups approach standard definition tasks differently. For instance, scholars and academics tend to set higher-level standards and administrators and institutions that are being accredited are inclined to propose lower-level standards which inherently creates a conflict of interest [47]. Consistent with our findings, evaluability is another issue for accreditation standards since the dominant perspective in this field is considering standards as agreed statements and principles instead of numerical indicators [28]. Hence, our findings regarding the optimization and evaluability were to be expected as we sent the validation survey for the administrations in medical schools that are being accredited. Further research is recommended to identify and compare the perspectives of different groups of UME stakeholders regarding the optimization and evaluability of standards.

We found that few written comments were reported for relevance which was consistent with the I-CVIs. Interestingly, a large number of written comments were on clarity while the I-CVI findings were adequate for this index. Considering the unsatisfactory results of I-CVIs for optimization and evaluability, we expected more comments for these two criteria. Kassebaum et al. (1998) conducted a national survey on the validity of LCME standards and the comments they received were mostly related to the clarity and evaluability of standards. They sent standards for different stakeholders of UME including the site visitors and conducted their survey after rounds of being tested in the accreditation process and these may explain the discrepancy with our finding regarding the evaluability index [41].

To improve the optimization and evaluability criteria, we held a participatory consultative workshop to meet the needs of varied stakeholder groups and to reach a consensus on challenging aspects of standards. Consensus on standards was achieved after overwhelming discussions on different viewpoints and convincing the last doubtful voice. As Galukande et al. (2013) reported subjecting the process to disputes is supposed to promote optimization and applicability of the standards and their ownership among stakeholders [48].

The national standards were based on a modification of the WFME standards, with the addition of Iranian UME programs’ specifications. We maintained the overall structure of WFME standards in terms of components and areas. However, the total number of components (subareas, basic and quality improvement standards, and annotations) decreased in national standards compared with WFME standards. The national standards also underwent many revisions in respect of the content. Areas such as ‘Students’ changed dramatically and we moved beyond WFME standards since issues such as students’ welfare are entirely well-established in our context. On the other side, the ‘Program Evaluation’ area, for instance, become easier as there was a little experience and reported practice in our context in this regard. The WFME team has not used specific model for developing program evaluation standards and they were basically focused on the triangulation of evaluation data. We followed their format, yet reduced the aspects of the UME programs that should be evaluated and the sources of data gathering (e.g. we removed the standard related to teacher feedback) in basic standards. All of these changes are supportive of the fitness of developed standards to the local context. Ho et al. (2017) explored the standards of three accreditation agencies in Taiwan, Japan and South Korea with their reference standards and concluded that each agency made adaptations compatible with its local context. They summarized the differences with reference standards in four categories of ‘Structural’, ‘Regulatory’, ‘Developmental’ and ‘Aspirational’ differences [49]. Further research is suggested comparing our national standards with WFME standards using the ‘difference’ categories.

The development of UME standards in a manner that reflects both local circumstances and international benchmarks provides a reasonable ground for the ‘glocalization’ of our accreditation system. Glocalization refers to accreditation that addresses both global and local demands [49]. This glocalization assures UME stakeholders, particularly the society as to the quality of UME programs and it may promote the UME program’s reputation at the international level which in turn, increases the rate of international applicants. Another advantage of glocalization is to achieve ‘Recognition’ by the WFME. The SCUME applied for WFME recognition in November 2017 and received the approved recognition status in June 2019 [30].

Finally, the national standards are to function as a lever for change and reform in the UME program in Iran within or outside of the accreditation by SCUME. They have been used by SCUME since 2017 in both self-study and external evaluation phases of accreditation. Future studies are suggested to identify the impact of defining standards in specific and establishing accreditation systems in general on UME programs using mixed methods. The proposed standards as well as our participatory approach to developing standards will be helpful in providing guidance for relevant institutions.

### Limitations

There are several limitations to this study. First, our method for developing and validating standards was mainly reliant on consultative workshops which may be prone to dominance effect and groupthink and lead to biased outcomes [50]. Although we made some efforts to mitigate this potential issue including informing participants about the ground rules of group discussion, recruiting experienced facilitators and diversifying the working groups’ composition, we cannot ensure its complete removal. Second, even though, we involved diverse stakeholders in different stages of standards development and validation, it may not be illustrative of all relevant stakeholders in UME in Iran. In particular, we missed representatives of scientific societies, graduates and patients. Another limitation would be the dominance of medical school administration as the end users of the ultimate standards throughout the validation study. We send the validation survey to UME directors and encouraged them to complete the survey after obtaining broader UME stakeholder views but we are not aware of the breath of involvement. We also invited other stakeholder groups including medical education experts for the subsequent consultative workshop to mitigate the end users’ dominance. Another limitation was that we performed a single round of the validation survey and refinement during the consultative workshop. Albeit, we considered several strategies to reach a consensus during the group discussions, conducting a revalidation round of validation survey after the refinement could have provided further evidence regarding the validity of standards, particularly for optimization and evaluability criteria. Additionally, we took a survey method and then a consensus approach to validate the standard set and we did not elicit stakeholders’ perceptions and concerns on standards through an in-depth individualized approach. Further studies are recommended to investigate the UME stakeholders’ views on standards after one run of implementation during accreditation assessment. Moreover, area 9 was developed by the task force and was not sent for validation which requires to be investigated in further study. Finally, more decline in the number of quality improvement standards in the final set may restrict the movement of medical schools in certain areas. Although this was our first experience for establishing an accreditation system in Iran and this was not unexpected as a result of ‘glocalization’, we should pay attention to this issue in the future revision of standards.

## Conclusion

Developing authentic accreditation standards that mirror both local features and international specifications requires adopting an approved set(s) of the international standard framework in a complicated iterative process of drafting and refinement involving many local stakeholders. We found the WFME BME, 2015 completely helpful as a benchmark to draft standards. We also observed that a consultative approach with the participation of a range of stakeholders and the recruitment of experienced facilitators could result in a set of standards compatible with UME local features. It is important to make a balance in the involvement of stakeholder groups including academics, administrators, trainees and graduates, medical education experts, patients and so on. We benefited greatly from the involvement of medical education professionals in refining the opinions of other groups. Using a validation survey with the participation of different stakeholders can be informative as an operating point for discussions of consultative workshops as well as for confirming the results of the discussions. We applied a validation survey with the former aim and invited only the director of the UME program. The results were satisfactory for relevance and clarity of standards yet inadequate for optimization and evaluability of standards. Further research is suggested with the involvement of more participant groups and several rounds of consensus achievement.

### Electronic supplementary material

Below is the link to the electronic supplementary material.

Supplementary Material 1

## Data Availability

All data generated or analysed during this study are included in this published article and its supplementary information files.

## Abbreviations

UME: Undergraduate Medical Education
LCME: Liaison Committee on Medical Education
MD: Doctor of Medicine
NCAAA: National Commission for Academic Assessment and Accreditation
IHPER: Institute of Health Professions Education and Research
ACGME: Accreditation Council for Graduate Medical Education
WFME: World Federation for Medical Education
BME: Basic Medical Education
CPD: Continuing Professional Development
AMEWPR: Association for Medical Education in the Western Pacific Region
SCUME: Secretariat of the Council for Undergraduate Medical Education
MoHME: Ministry of Health and Medical Education
I-CVI: content validity index at the item level
CVI: content validity index
