# Supplementary material for: Developing and validating a national set of standards for undergraduate medical education using the WFME framework: the experience of an accreditation system in Iran
Source: BMC Med Educ. 2023 May 24;23:379. doi: 10.1186/s12909-023-04343-9 (PMC10210375; doi:10.1186/s12909-023-04343-9)
Supplement: Supplementary file 1 — Supplementary Material 1 [file 12909_2023_4343_MOESM1_ESM.docx]

Online Supplemental Appendix 1

NATIONAL STANDARDS FOR UNDERGRADUATE MEDICAL EDUCATION PROGRAM OF ISLAMIC REPUBLIC OF IRAN (*Edition: 2017)*

**Area 1: MISSION AND OBJECTIVES**

- 1. **MISSION AND OBJECTIVES**

**Basic Standards**

The medical school **must**:

- - 1. Formulate its Undergraduate Medical Education Program mission in accordance with community health needs, health services system provision, upstream documents, values and social accountability.
    2. Formulate its mission by collaboration with the principal stakeholders.
    3. Set its goals and educational strategies in such a way that implementation of the program would lead to training of competent graduates capable of undertaking the roles of General Practitioner and professional development via lifelong learning and well prepared and ready for postgraduate medical education (if inclined to).

**Quality Development Standards**

The medical school **should**:

- - 1. Ensure that in formulation and revision of its mission employs a wide range of opinions of other stakeholders.
    2. Considers new medical achievements and global aspects of health in its mission statement.

**Annotations:**

- Community health needs, and health system services are determined on the basis of official reports of the Ministry of health, and Medical education, in particular the reports of vice ministers for health and treatment.
- Upstream documents include all national documents relating to Undergraduate Medical Education Program such as health general policy (communicated by Supreme Leader) Iran Comprehensive Scientific Map, Health documents, Educational Evolution documents, and expected competencies of Medical doctors
- The values include transcendent Islamic values in line with Divine Satisfaction, human munificence , honor to serve humans, necessity to maintain life, population revival, promotion of health and quality of life, equity in health, importance of pain relief in sufferers, commitment to divine commandments, human dignity, obligation to medical sentences, respecting human rights, knowledge seeking, necessity of medical profession, provision and warranty of priorities diagnosed and declared by health services system and having required flexibility to follow jurisprudence requirements, and developing and teaching emerging medical issues.
- Consider related aspects of social accountability in relation to Undergraduate Medical Education Program.
- Lifelong learning means that students contribute in to his/ her learning and emerging knowledge and personal competency in accordance with community needs and scientific-technological changes in medicine.
- The principal stakeholders mean university top managers, dean and authorities of medical school, faculty members, students and graduates.
- Other stockholders mean representative of other medical professions, related organizations (Medical Council, insurance companies) and community members (health services receivers), National Undergraduate Medical Education Program Board, and related authorities in the Ministry of Health and Medical Education.
- A competent Medical Doctor is a person who is capable of independent practice and plays roles of General Practitioner on the basis of the last edition of competency framework of Medical Doctor approved by Health Sciences Higher Council of Planning.
- New medical achievements are medical innovations including innovation equipment, techniques, medical knowledge and medical education.
- Aspects of global Health primarily include regional priorities and global health issues.
  1. **AUTONOMY**

**Basic standards**

Medical school **must**:

- - 1. Ensure institutional autonomy to design and implement Undergraduate Medical Education Program, and use of allocated resources necessary for implementation of the program.

**Area 2: EDUCATIONAL PROGRAM**

- 1. **FRAMEWORK OF THE PROGRAM**

**Basic Standards**

The medical school **must**:

- - 1. Develop its educational program on the basis of the national framework and curriculum content for Undergraduate Medical Education Program communicated by the Ministry of Health and Medical Education, be approved by the university educational council and communicate the curriculum by appropriate media such as the school official Website.
    2. Indicates its endeavor to include some aspects of competency based education in formulation and implementation of Undergraduate Medical Education Program.
    3. Formulate and implement Undergraduate Medical Education Program on the basis of principles of educational equity.

**Quality Development Standards**

Medical school **should**:

- - 1. Formulate and implement its Undergraduate Medical Education Program in accordance with approved national medical doctors ’competency framework in such a way that could ensure its graduates competencies. To achieve this goal, it is necessary that all the elements of the program be planned and implemented based on approved competencies

**Annotations:**

- The school of medicine educational program for Undergraduate Medical Education Program includes the major components of any educational program including competencies (an integration of knowledge, skills and attitudes the students will achieve), content and learning experiences, teaching and learning methods, student assessment and program evaluation.
- Principles of educational equity mean equal treatment of students and faculty members irrespective of gender, ethnicity, religion, socioeconomic status and physical capabilities on the basis of the minimal requirements and according to the existing regulations.
  1. **EDUCATIONAL CONTENT**

**Basic standards**

The school of medicine **must**:

- - 1. Incorporate the core contents of biomedical sciences mentioned in national framework and curriculum content which is necessary for medical practice including facts, concepts and principles required for acquiring and practicing clinical sciences.
    2. Incorporate the core contents of clinical sciences mentioned in national framework and curriculum content which is necessary for medical practice, including professional and clinical knowledge and skills, required for independent medical practice after graduation as a medical doctor in the country.
    3. Incorporate the contents related to social and behavioral sciences, medical ethics, professionalism, medical jurisprudence, complementary medicine and in particular traditional medicine required for medical doctors.
    4. Incorporate content of the general courses required for training of medical doctors in its educational program.
    5. Incorporate scientific methods required for medical doctors’ practice in its educational program including critical thinking, research and Evidence Based Medicine or BEME.

**Quality Improvement Standards**

The medical school **should:**

- - 1. Monitor and modify the educational contents (basic sciences, clinical sciences, social and behavioral sciences and scientific methods) in accordance with current and anticipated needs of the community, scientific and technological advances, within the national curriculum framework and without increasing the overall content of the curriculum.

**Annotations:**

- Core content includes the educational program content which all students must learn.
- Basic sciences include disciplines such as anatomical sciences (anatomy, histology and embryology) biochemistry, physiology, biophysics, genetics, immunology, microbiology (bacteriology, parasitology, virology) molecular biology and pathology.
- Behavioral and social sciences include disciplines such as community medicine, epidemiology, biostatistics, global health, medical psychology, medical sociology, public health and social sciences.
- Clinical Sciences include disciplines such as internal medicine, general surgery, pediatrics, gynecology, psychiatry, emergency medicine, dermatology, orthopedics, urology, ophthalmology, ENT, palliative medicine, radiology, and occupational medicine.
- Complementary medicine includes traditional and alternative medicine.
- Clinical skills include history taking, physical examination, communication skills, and diagnostic and therapeutic procedures.
- Factual knowledge is the proved or recognized factual knowledge in medical education is memorized for future recall. Concepts are used to simplify the world and organize the phenomena. Principles explain the relationship between the concepts.
- General courses include Islamic education, Islamic moral, Islamic texts, Persian literature, English language, physical training, etc.
- Scientific method is an integrated process of inductive and deductive reasoning in which the researcher presents hypothesis, based on his observation, in an inductive way and then by applying deductive reasoning logically applies the hypothesis.
- Medical practice means all the expected roles and tasks mentioned in national competency frameworks for Medical Doctors.

- 1. **EDUCATIONAL STRATEGIES**

**Basic standards**

The medical school **must**:

- - 1. Implement some degrees of horizontal integration of related sciences.
    2. Implemented part of its educational program in accordance with community based education in inpatient and outpatient fields with a comprehensive health oriented approach.

**Quality development standards**

The medical school **should**:

- - 1. Incorporate vertical integration of clinical sciences, biomedical sciences, behavioral and social sciences and other contents related to Undergraduate Medical Education Program.
    2. Incorporate electives on the basis of national curriculum and resources of the universities.
    3. Develop and implement Undergraduate Medical Education Program to achieve student centeredness and lifelong learning.

**Annotations:**

- Horizontal integration (concurrent) means integration of basic biomedical courses such as anatomy, physiology or integration of clinical sciences such as internal medicine and surgery or nephrology and urology.
- Vertical integration (linear) means integration of courses at different stages such as biochemistry and metabolic diseases or physiology and nervous system.
- Elective contents are a part of educational program and students can select them according to their personal interests or needs for deeper learning.
- For lifelong learning see mission and goals.
- Student centeredness means provision of learning opportunities with the goal of shifting the learning responsibilities to the students and changing them to independent self-directed learners. In this framework, students’ collaboration is crucial for learning.
  1. **TEACHING AND LEARNING METHODS**

**Basic standards**

The medical school **must**:

- - 1. Apply teaching methods compatible with educational objectives to achieve expected competencies.
    2. Improve the quality and increase the interaction with students in traditional teaching methods such as lectures.
    3. Provide specific learning opportunities in clinical rotations outside and inside hospital environments in accordance with the needs of medical doctors.
    4. Provide the conditions in which students collaborate with health care provider team, while accepting gradual responsibility, enjoying suitable supervision in accordance with their levels and receiving ample feedbacks.

**Quality development standards**

The medical school **should**:

- - 1. apply active teaching methods such as team based learning, case-based learning, problem-based learning, learning in small groups, electronic learning, simulation-based learning and other active methods in educational content presentation and in accordance with educational competencies and objectives.

**Annotations:**

- Accepting gradual responsibility means that the students as observers gradually become colleagues and active agents in learning process in clinical settings.
- Specific educational opportunities are planned educational opportunities which are presented on the basis of the time and expected objectives.

**Area 3: STUDENT ASSESSMENT**

**Basic standards**

The medical school **must**:

- - 1. Develop, announce and implement student assessment system.
    2. Ensure that student assessment covers all aspects of competencies including cognitive, psychomotor (skills) and affective (attitudes) domains.
    3. Use a wide range of appropriate assessment methods according to objectives and expected competencies of students and utility of assessment tools.
    4. Ensure that specific mechanisms are used to report assessment results and feedbacks to students’ appeal of assessment.
    5. Feedback students on the basis of summative assessments.
    6. Review and document the analysis (quantitatively or quantitatively) of multiple choice exams and give feedback to test developers on the basis of the analyses.
    7. Ensure that the processes and documents of administered tests are open to scrutiny by external expertise.

**Quality development standards**

Medical schools **should**:

- - 1. Analyze the results of other tests qualitatively and quantitatively (except MCQs) and provides test developers with feedbacks on the basis of the results.
    2. Ensures that there is a documented plan to monitor the quality of tests and that it is implemented.
    3. Organize formative assessments and provides students with continuous effective feedbacks.

**Annotations:**

- Assessment system should include principles and objectives of assessments, assessment tools, schedules, standard setting methods, giving feedback, test security, allowed retake mechanism, ethical considerations (examiners’ conflict of interest avoidance methods) in different phases of Undergraduate Medical Education Program.
- Different aspects of competencies include cognitive aspects as well as aspects such as clinical care, ethical and professional behaviors and communication skills.
- Regarding the complexity of outcomes in Undergraduate Medical Education Program, a single assessment tool is not sufficient for assessment and diverse tools should be used for assessing cognitive, affective and psychomotor domains. These tools include written exams (open ended, close ended), oral exam, Objective Structured Clinical Examinations (OSCE), Direct Observation of Procedural Skills (DOPS), Mini clinical Evaluation Exercise (Mini-CEX), portfolio, logbook, 360-degree assessment and other valid assessments methods and tools.
- Assessment utility is the combination of validity, reliability, educational impact, acceptability and efficiency of the assessment tools in different situations. These criteria are assessed via statistical and qualitative methods.
- Feedback not only means announcing the scores, but also announcing detailed key to the questions, identifying students’ mistakes and providing advice for performance improvement
- Formative assessment means tests that are administered to make decisions about students’ performance. Qualitative and Quantitative analysis of tests mean evaluating each question and determining the accuracy level and flaws before (according to the respective check list via review session with peers and test experts) or and after the exam (determination of difficulty and differentiation coefficient and other related indices). In addition to analysis of each question and item, it is necessary to determine utility of each student assessment tool or method and student assessment program global.
- External experts are external faculty or university experts or experts from international organizations.
- Formative assessments are those tests administered during the course for giving feedback and improving student performance but the results are not considered in the final assessment.

**Area 4: STUDENTS**

- 1. **ADMISSION POLICY AND STUDENT SELECTION**

**Basic standards**

The medical school **must**:

- - 1. Formulate and implement a specific program to assess characteristics of admitted students and while using the results of this assessment in future policy making for students and continuously submitting them to top rank authorities.
    2. Formulate and implement a specific program for determining the school admission capacity within the current framework of upstream rules and policies and on the basis of appropriate evidence to determine school capacity, and student admission.
    3. Have a plan to introduce Undergraduate Medical Education Program, and school capacity to new students. In addition, orientation of new students to educational, professional and disciplinary regulations, and expected professional tasks should be considered in this plan.

**Quality development standards**

The medical school **should**:

- - 1. Have a plan to introduce the discipline of medicine and school capacity to high school students of the areas of service.
    2. Provide feedbacks to organizations responsible for student selection to conform with changes in Undergraduate Medical Education Program and the balance between the number of admitted students and school capacities, and to revise respective policies.

**Annotations:**

- The program related to new students would include the followings:
- Physical, mental, social, spiritual health appropriate for medical profession
- Tendency to acquire individual merits, growth and excellence
- Appropriate awareness and attitudes toward educational responsibilities, roles and socio-professional status of medical doctors.
- Basic general competencies for appropriate performance in medical profession would include skills in:
- Communication
- Decision making and critical thinking
- Team work
- Leadership and management
- Personal managements
- Logical reasoning
- Reflection
- Creativity
- Geographic and demographic distribution of new students
- Academic performance before university entrance Including GPA (Grade Point Average) at high-school, student performance in university entrance examination, attendance at student Olympiads and other academic achievements (e.g. attendance at student festivals).
- Appropriate evidence to determine student admission capacity and continuous revision and promotion of the programs related to student admission may include the followings:
- Upstream documents and policies
- Human resources of the school including, faculty members, staff, and managers
- Capital resources of school including physical facilities of the school, hospitals, etc.
- Consuming resources of school including cash budget, equipment and facilities
- Societal needs (in particular in the respective province and city of the school)
- Monitoring of the effectiveness of student admission capacity in the past years
- School capacity means, learning opportunities / strengths such as physical resources, learning opportunities, educational departments, etc.
  1. **STUDENT SUPPORT AND CONSULTATION**

**Basic Standards**

The medical school **must**:

- - 1. To design and implement an appropriate program to support social, legal, ethical, and mental safety of the students, and to protect them against potential harms.
    2. To design and implement an appropriate system to provide educational and mental counseling services for students and document the results while ensuring the confidentiality of the information. This system should actively identify the students in need of these services and monitor them. In addition, the students should have the access to the counseling center of the school.
    3. To design and implement extra-curricular activities to facilitate personal, characteristic, cultural, social, and spiritual growth, as well as increasing academic motivation and vitality and professional conduct.
    4. Identify the minimum requirements of welfare for undergraduate medical students and present a plan for provision and sponsorship.
    5. Have a specific mechanism to identify and promote professional behaviors in students and to treat inappropriate behaviors while considering current regulations.
    6. To design and implement a specific program to support a cohort of problem students.
    7. To design and implement specific programs to coordinate respective units in order to support organized scientific-cultural activities of students, in the framework of upstream regulations and policies.

**Quality development standards**

School of medicine **should:**

- - 1. Employ eligible students in addition to faculty members and experts, for providing counseling and mentorship services in the program of educational and mental counseling services to students.
    2. Monitor the educational and mental counseling services provided to the students and examine its effectiveness.
    3. Continuously revise and promote programs related to student support standards.

**Annotations:**

- An appropriate system for providing educational and mental counseling services to students includes individuals, rules and regulations, and processes which could be achieved in the framework of a specific institution of the school or in collaboration with other institutions.
- Welfare support includes:
- Dormitory
- Student loan
- Nutrition
- Health insurance
- Sport and leisure facilities and spaces
- Extra-curricular plans
- Student work inside and outside of the university
- Access to information technology
- Plans for student transportation and accommodation in university and hospital settings.
- A student cohort includes:
- Gifted and talented students
- Students with academic underperformance
- Students with specific physical or mental needs
- Students with inappropriate economic and welfare conditions
- Supports could be provided by the activities of the institution in charge or by cooperation of several institutions. In all circumstances, it is recommended to preserve integrity, transparency, and accountability of the institutions at the highest coordinated level.
- Promotion and review of programs related to student support standards must be on the basis of the below evidence:
- Upstream documents and policies
- School and societal needs
- Student needs
- Student demands
- Student satisfaction of the received supports
- School resources
- Eligible students mean those who are scientifically, ethically, and behaviorally approved by the school.
  1. **STUDENT REPRESENTATIVES**

**Basic standards**

The medical school **must**:

- - 1. Provide the required context for organized participation of students in policy-making and administrative processes related to Undergraduate Medical Education Program.

**Quality development standards**

- The medical school **should**:
  - 1. All the programs related to presence of students' representatives should be revised and promoted continuously to the utmost possible point leading to the process of student collaboration promotion.

**Annotations:**

- Student participation in a process refers to their representatives’ roles in planning, implementing and evaluation of that process. This could change from active consultation roles to permanent membership in decision-maker institutions of the school.
- Continuous revision and promotion of the programs related to standards for student representatives’ presence should be based on the following evidence:
- Upstream documents and policies
- School needs
- Societal needs
- Students’ and faculties’ demands
- Student satisfaction of their level of participation

**Area 5: ACADEMIC STAFF/FACULTY**

- 1. **RECRUITMENT AND SELECTION OF FACULTY**

**Basic standards**

The medical school **must:**

- - 1. Formulate and implement faculty recruitment policies
    2. Provide a recruitment program on the basis of Undergraduate Medical Education Program and the number of required biomedical, clinical, social and behavioral sciences faculty. In this program, the proportion of geographic full-time, full-time, and part-time faculty, the proportion of medical and non-medical faculty members and the proportion of faculty members and students be considered.
    3. Recruitment program should be based on the scientific/practical, educational, and research competencies, ethics and professional conduct.
    4. Develop a continuous monitoring and evaluation program on the basis of duties and responsibilities assigned to faculty members.

**Quality development standards**

The medical school **should**:

- - 1. Have a recruitment program in-line with mission of the school, considering regional specifications.
    2. In the recruitment program considers the school budget and proprietary income.

**Annotations:**

- Faculty recruitment and selection means ensuring efficiency of faculty of basic, clinical, social and behavioral sciences in the areas of education, research and delivery of clinical services and on the basis of Undergraduate Medical Education Program.
- Number, diversity, proportion and adjustment of faculty selection and recruitment mean meeting the nationwide basic standards of the country.
- Scientific/practical, educational, research, ethical and professional conduct competencies are in accordance with criteria of regulations for faculty recruitment issued by the Higher Council of Cultural Revolution.
- Regional specifications are those mentioned in the Territory Survey Document.
- Evaluation of the prospective faculty members is based on the applicants’ CV and regulations for faculty recruitment.
- Monitoring and evaluation of faculty members in the process of promotion is based on academic (teaching, research and service experiences) CV, and achievements in the last years before he applies.
  1. **FACULTY PROMOTION AND ACTIVITIES**

**Basic standards**

The medical school **must**:

- - 1. Provide and implement a program that ensures a balance between different activities of faculty members on the basis of employment responsibilities and promotion regulations.
    2. Ensures maintenance of educational, research, cultural competencies and health services delivery of the faculty members.
    3. Provide and implement a program that ensures the application of clinical and research activities in teaching and learning process.
    4. Have a program to empower and support faculty members on the basis of faculty promotion regulations.
    5. Ensures that faculty members are sufficiently familiar with Undergraduate Medical Education Program curriculum and spend enough time and attention to the Undergraduate Medical Education Program tasks assigned to them.
    6. Ensures that there is a program to motivate and empower faculty members in student assessment.
    7. Ensures that there is a program to provide minimum welfare for the faculty members/instructors.

**Annotations:**

- Educational, research, cultural competencies and service delivery are defined in terms of regulations for faculty promotion.

**Area 6: EDUCATIONAL RESOURCES**

- 1. **PHYSICAL FACILITIES**

**Basic standards**

The medical school **must**:

- - 1. Provide sufficient and suitable physical facilities for faculty members and students to ensure the educational program will be implemented appropriately.
    2. Provide learning environment in which safety principles are considered for staff, students, patients and relatives.
    3. Improve student learning environment by updating and extending facilities regularly and in accordance with the current advances.

**Annotations:**

- Physical facilities include lecture halls, classrooms, team work rooms, research and educational laboratories, clinical skill labs, administrative offices, libraries, information technology facilities, exam halls, restaurant, gyms, leisure facilities, transportation, residency places (dormitory and pavilions).
- The minimum requirement of the physical facilities is specified in the documents of Undergraduate Medical Education Program standards.
- A safe environment (work environment safety) includes provision of necessary information and protection against harmful materials, specimens, regulations and safety of laboratories and equipment.
  1. **CLINICAL EDUCATION RESOURCES**

**Basic standards:**

The medical school **must**:

- - 1. Ensure the provision of the following items to acquire ample clinical experience by the students:
- Sufficient number and appropriate variations in patients
- Clinical education facilities
- Clinical education supervision

**Quality development standards:**

The medical school **should:**

- - 1. Provide and evaluate clinical education facilities to meet the needs of the population it serves.

**Annotations:**

- Patient in clinical education means the real patient, but in necessary cases simulated patients, standard patients or other techniques could be replaced.
- The required clinical education facilities are specified in the documents of Undergraduate Medical Education Program standards.
- Clinical education facilities include an appropriate combination of hospitals type 1, 2, and 3, out-patient services, clinics, primary health care centers, health care centers, and other health care centers as well as clinical skill labs.
- Evaluation of clinical education facilities includes regular evaluation of Undergraduate Medical Education Program, along with evaluation of quality of facilities, number and variations in patients, therapeutic measures, supervision and their management.
  1. **INFORMATION TECHNOLOGY**

**Basic standards**

The medical school **must**:

- - 1. Develop and implement policies for the effective and ethical use of information technology facilities by students and faculty members.
    2. Provide easy access to electronic media and network connections for faculty members and students.
    3. Provide faculty members and students with access to information technology by determining the level of access and ethical principles of professionalism for the followings:
- Patient management
  - Access to patient information
- Work in health care systems
- Clinical skills education

**Quality development standards:**

The medical school **should**:

- - 1. Provide facilities and modern educational technology equipment such as advanced simulators, and virtual reality to teach medical students.

**Annotations:**

- The policy for effective and ethical use of information technology and communications includes computer, telephone, mobile, intra and external networks and other tools; connectivity to library services including sharing the resources and educational materials via a learning management system as well as information technology. The use of information technology may be part of best evidence medical education and preparing students for life-long learning ad continuous professional development.
- Ethical use refers to challenges related to physicians and patient privacy, confidentiality of patient information when encountering the advances of information technology in medical education and health care. Policies related to empowering the physician and the patient to make the best use of the new technology would be helpful.
  1. **RESEARCH AND SCHOLARSHIP**

**Basic standards**

The medical school **must**:

- - 1. Provide the required research infrastructures for implementation of Undergraduate Medical Education Program for medical students.
    2. Have specified research facilities and priorities and inform faculty members and students.
    3. Enforce the connection between education and research by developing and implementing specified policies.
    4. Consider medical research and scholarship as the basis of modification for educational program.

**Quality development standards**

The medical school **should**:

- - 1. Ensures that medical research and scholarship affect the current education via facilitating learning of scientific methods and evidence based education.

**Annotations:**

- Medical research and scholarship include scientific research in basic, clinical, behavioral and social sciences. Medical scholarship means to achieve advance medical knowledge and research. Medical research could be the basis of educational programs.
  1. **MEDICAL EDUCATION EXPERTISE**

**Basic standards**

The medical school **must**:

- - 1. have access to educational expertise.
    2. Formulate and implement a policy on the use of educational expertise in development of teaching and assessment methods and empowerment of faculties.

**Quality development standards:**

The medical school **should**:

- - 1. Pay attention to current expertise in medical education research.
    2. Have medical education experts at the university level and have access to national and international expert consultation.

**Annotations:**

- Medical education experts include individuals with developmental and research activities from different health professions (e.g. physicians, nurses, pharmacists, dentists, etc.), educational experts (medical education graduates, psychologists, sociologists, curriculum planners, evaluation expert, etc.).
- Research in medical education surveys the effectiveness of different aspects of teaching and learning.
  1. **EDUCATIONAL EXCHANGES**

**Basic standards**

The medical school **must**:

- - 1. Formulate a policy for national and international collaboration with other educational organizations such as student and faculty exchanges, and transfer of educational credits from other organizations.

**Quality development standards**

The medical school **should**:

- - 1. Facilitate Faculty/ instructors, students and staff exchange at regional and international levels by providing appropriate resources.

**Annotations:**

- Other educational institutions would include other medical schools as well as other.
- Faculties and educational institutions (e.g. dentistry, public health and veterinary medicine) and other health professions.
- Staff would include administrative and technical personnel.
- Transfer of educational credits would be facilitated via coordination between medical schools.

**Area 7: PROGRAM EVALUATION**

- 1. **PROGRAMME EVALUATION AND MONITORING**

**Basic standards**

The medical school **must**:

- - 1. Formulate and implement a program for systematic evaluation of Undergraduate Medical Education Program.
    2. Formulate and implement a program for monitoring processes and outcomes of Undergraduate Medical Education Program.
    3. Employ the results of evaluation for revising Undergraduate Medical Education Program.
    4. Engage the main stakeholders of evaluation in monitoring and evaluation activities of the educational program.

**Quality development standards**

The medical school **should**:

- - 1. Periodically (at least every 5 years) evaluate other aspects of Undergraduate Medical Education Program comprehensively.

**Annotations:**

- Program evaluation is the process of systematic gathering and analysis of information to judge the effectiveness and adequacy of the educational program and its core elements (including curriculum model, program structure, components and length of the course, elective and core courses). Data collection is performed with different data gathering methods such as questionnaires, group discussions, interviews, document surveys, and with valid and reliable tools and from different resources such as students, teachers and program managers.
- Programmed monitoring would include the continuous collection of data about key aspects of the curriculum to ensure the verification of the educational process and identification of any areas in need of intervention. Educational processes are activities which are implemented to achieve intended educational outcomes. Educational outcomes are defined as short, mid and long-term outcomes of the program.
- Other aspects of educational program are the resources, structure, educational setting, dominant culture, long-term outcomes, particularities of the course (e.g. specific educational and evaluation methods) and social accountability.
- The main stake-holders (see annotations of mission and objectives).
  1. **STUDENTS AND GRADUATES’ PERFORMANCE**

**Basic standards**

The medical school **must**:

- - 1. Analyze the performance of all students in relation to intended educational outcomes and educational program.

**Quality development standards**

The medical school **should**:

- - 1. Analyze the performance of all students in relation to their conditions, backgrounds, and required entrance competencies.
    2. Provide the results of students’ and graduates’ performance analysis and give feedbacks to the respective authorities such as ministry of health, testing organization, curriculum planning committee and student counseling.

**Annotations:**

- Student performance analysis means assessing the length of education, scores, the rate of pass and fail, the rate of success or conditional pass and the reasons, academic status of the students on the basis of self-report, personal interests, including elective courses, and interview with students failing the course, quitting the course and dismissed, etc.
- Graduates performance analysis means assessing the results of performance in national tests, occupation, and post-graduation performance, etc.

**Area 8: GOVERNANCE AND ADMINISTRATION**

- 1. **GOVERNANCE**

**Basic standards**

The medical school **must**:

- - 1. Receive the establishment permission issued by the council of expansion of medical universities in ministry of health and medical education.
    2. To achieve the objectives of Undergraduate Medical Education Program, define, develop, implement and monitor its structure, organizations, authorities, responsibilities, and intra-organizational communications.
    3. Use structure of councils with specific defined duties (for decision and policy making) and structure of committees (for technical and administrative activities) with collaboration of main stakeholders in governance.

**Quality development standards**

The medical school **should**:

- - 1. Inform the stakeholders about governance decisions and performances transparently.

**Annotations:**

- Governance structure: includes board of directors, policy-making council, and program and institutional policy establishment, implementation and monitoring processes.
- Committee structure means a structure for implementation of Undergraduate Medical Education Program and policies such as curriculum planning committee, recruitment committee, etc.
- Principal stakeholders (see annotations of mission and objectives)
- Program and institutional policies include missions of medical school, educational program, admission system, personnel recruitment, policy selection, effective decision making in interaction and communication with health and clinical sector.
  1. **EDUCATIONAL MANAGEMENT**

**Basic standards:**

The medical school **must**:

- - 1. Appoint Undergraduate Medical Education Program authorities and define and formulate their administrative tasks.

**Quality development standards**

The medical school **should**:

- - 1. Have a specific plan for performance evaluation of administrative team and periodically evaluate and monitor the team performance in regard to achievement of intended mission and outcomes.

**Annotations:**

- Educational administration team: real and legal individuals of board of directors, and governance structure accountable in regard to educational, research, service and decision-making issues. These individuals include dean of medical school, vice-dean for biomedical education, vice-dean for clinical education, the committee for implementation of biomedical sciences standards, head of educational departments, educational committees of educational departments (basic and clinical sciences), head of educational sections (basic and clinical), presidents and vice-president for educational affairs of teaching hospitals.
  1. **EDUCATIONAL BUDGET AND RESOURCE ALLOCATION**

**Basic standards**

The medical school **must**:

- - 1. Have a specified budget for achieving missions and implementing Undergraduate Medical Education Program.
    2. Have authority to allocate budget and administrative power to achieve and develop activities related to Undergraduate Medical Education Program on the basis of standards.

**Annotations:**

- Specified budget: It means educational budget is based on the budgetary transactions of the school which should be reimbursed from a transparent and specified part of medical school budget.
  1. **MANAGEMENT AND ADMINISTRATION**

**Basic standards**

The medical school **must**:

- - 1. Have professional administrative staff necessary for implementation of educational program.
    2. Formulate and implement a specific program for provision of human, monetary and consuming resources required for continuity and promotion of student activities in collaboration with vice chancellor for cultural and student affairs in the following areas:
- Students;
- Religious, cultural and sport activities
- Scientific activities
- Individual growth and social activities
  - 1. Have a specific structure and authority to manage student assessment process, and continuous evaluation for improvement and quality promotion of student assessment.

**Annotations:**

- Professional administrative staffs are experts matched with needs of the school including financial managers, budget staff, educational and research assistants, etc.
  1. **INTERACTION WITH HEALTH SECTOR**

**Basic standards**

The medical school **must**:

- - 1. Have a specified and appropriate process for continuous constructive interaction with health sector (health and treatment, food and drug) and the corresponding social organizations.

**Annotations:**

- Constructive interaction includes information exchange, collaboration, and cooperation with respective organizations, to educate accountable physicians for the future needs of the society.

**Area 9: CONTINUOUS RENEWAL**

**Basic standards**

The medical school **must:**

- - 1. Assess and revise Undergraduate Medical Education Program and its components continuously.
    2. Take actions to remove identified weaknesses of Undergraduate Medical Education Program and its components.
    3. Allocate an appropriate budget for continuous renewal of Undergraduate Medical Education Program.

**Quality development standards**

The medical school **should**:

- - 1. Base the process of continuous renewal on the results of prospective studies, evaluation and medical education literature review.

Address the followings issues in the process of continuous renewal:

- - 1. Revision of its mission statement in accordance with societal, scientific, economic and cultural reforms.
    2. Renewal of competencies for graduates in accordance with the needs of future work settings.
    3. Renewal of educational planning models and methods to ensure their appropriateness.
    4. Revision of educational contents in accordance with scientific achievements in the areas of biomedical, clinical, social, behavioral sciences, and changes in demographics and epidemiology characteristics by introducing new cases and removing old issues from the educational program.
    5. Quality improvement of student assessment programs, and methods and number of tests on the basis of the changes in outcomes of learning and teaching methods.
    6. Feedback to institutions related to student admission for improvement of the policies and conformity with Undergraduate Medical Education Program changes and proportion of the number of the selected students with faculty capacity.
    7. Revising the faculty recruitment process according to the emerging needs of the school.
    8. Renovation of educational facilities and equipment according to the emerging needs including student selection capacity, the number and characteristics of faculty members and features of educational programs.
    9. Revision in evaluation and monitoring system of the Undergraduate Medical Education Program.
    10. Modification and development of the organizational structure and of governance, higher management and administrative organization of Undergraduate Medical Education Program for appropriate encounter with changing conditions and needs, as well as considering different stakeholders’ interests.

**Annotations:**

- Undergraduate Medical Education Program components mean mission and objectives, structure, processes, outcomes, content, teaching and learning methods, assessment, evaluation and learning environment.
